# Protective Effects of 6,7,4′-Trihydroxyflavanone on Hypoxia-Induced Neurotoxicity by Enhancement of HO-1 through Nrf2 Signaling Pathway

**DOI:** 10.3390/antiox10030341

**Published:** 2021-02-24

**Authors:** Hyun-Su Lee, Gil-Saeng Jeong

**Affiliations:** College of Pharmacy, Keimyung University, 1095 Dalgubeol-daero, Daegu 42601, Korea; hyunsu.lee@kmu.ac.kr

**Keywords:** 6,7,4’-trihydroxyflavanone, cobalt chloride, hypoxia, protection, neurotoxicity, nuclear factor erythroid 2-related factor 2 pathway

## Abstract

Since hypoxia-induced neurotoxicity is one of the major causes of neurodegenerative disorders, including the Alzheimer’s disease, continuous efforts to find a novel antioxidant from natural products are required for public health. 6,7,4′-trihydroxyflavanone (THF), isolated from *Dalbergia odorifera*, has been shown to inhibit osteoclast formation and have an antibacterial activity. However, no evidence has reported whether THF has a protective role against hypoxia-induced neurotoxicity. In this study, we found that THF is not cytotoxic, but pre-treatment with THF has a cytoprotective effect on CoCl_2_-induced hypoxia by restoring the expression of anti-apoptotic proteins in SH-SY5y cells. In addition, pre-treatment with THF suppressed CoCl_2_-induced hypoxia-related genes including *HIF1α*, *p53*, *VEGF*, and *GLUT1* at the mRNA and protein levels. Pre-treatment with THF also attenuated the oxidative stress occurred by CoCl_2_-induced hypoxia by preserving antioxidant proteins, including SOD and CAT. We revealed that treatment with THF promotes HO-1 expression through Nrf2 nuclear translocation. An inhibitor assay using tin protoporphyrin IX (SnPP) confirmed that the enhancement of HO-1 by pre-treatment with THF protects SH-SY5y cells from CoCl_2_-induced neurotoxicity under hypoxic conditions. Our results demonstrate the advantageous effects of THF against hypoxia-induced neurotoxicity through the HO-1/Nrf2 signaling pathway and provide a therapeutic insight for neurodegenerative disorders.

## 1. Introduction

Since continuous provision of enormous amounts of oxygen to the brain is required for its proper function, the brain is easily affected by the limited oxygen condition called hypoxia. Neuronal cytotoxicity is generally induced under hypoxic condition because insufficient supply of oxygen to the brain enhances the mortality and disability of neurons [1,2]. It has been reported that hypoxia-induced neurotoxicity causes brain damage and leads to neurodegenerative diseases, including Alzheimer’s disease, vascular dementia, and Parkinson’s disease [3,4].

Hypoxia inducible factor 1α (HIF1α) has been elucidated as the representative transcription factor of hypoxic condition that can be accumulated in cerebral cortex and hippocampus [5,6]. Several studies have reported that HIF1α induces the expression of various genes associated with cell survival, angiogenesis, or glucose uptake, including p53, vascular endothelial growth factor (VEGF), and GLUT1 to overcome hypoxic challenges [7,8,9]. These altered microenvironments promote oxidative stress by generating reactive oxygen species (ROS) in neuronal cells, leading to neuronal toxicity. Therefore, the importance is gradually elevated to develop a modulator in the neurotoxic condition induced by hypoxia.

Most cells have developed an endogenous self-defense strategy against ROS-induced damage. One of the most widely studied mechanisms is the promotion of heme oxygenase-1 (HO-1) expression via the nuclear transcription factor erythroid 2-like factor 2 (Nrf2) pathway [10,11]. The cytoprotective role of Nrf2 pathway has been reported to significantly reduce the cytotoxicity under hypoxic conditions [12]. Nonetheless, the protective effect of HO-1 via the Nrf2 pathway is important, and little evidence has been reported on whether bioactive molecules isolated from natural products induce the HO-1/Nrf2 pathway to reduce neurotoxicity induced by hypoxia.

6,7,4′-trihydroxyflavanone (THF), isolated from *Dalbergia odorifera*, is a flavonoid categorized in the flavanone family. *D. odorifera* has been studied to mainly live in Southern China, including Hainan, Fujian, Guangdong, and Zhejiang [13]. In East Asia, including Korea and China, *D. odorifera* extract has long been used as a therapeutic agent for rheumatic and epigastric pain, blood stagnation syndrome, ischemia, and swelling [14]. Accumulating evidences demonstrate that THF exhibits effective regulation of osteoclastogenesis by controlling bone resorption and protective role against methamphetamine-induced cytotoxicity on T cells [15,16]. In particular, it has been reported that THF effectively suppresses the NF-κB pathway in two studies to possess anti-osteoclastogenesis and cytoprotective effect against methamphetamine exposure. However, naringenin, one of the flavanone that has a similar structure with THF, has been elucidated to have a radical scavenging activity and protect liver tissue by acting as antioxidant [17,18], no evidence has been reported if THF possesses an antioxidant effect in CoCl_2_-induced hypoxia condition. In this study, we investigated whether treatment with THF has a cytoprotective role by inducing HO-1 expression through the Nrf2 pathway. In addition, we also explored whether induction of HO-1 by pre-treatment with THF attenuates hypoxia and oxidative stress to suppress neurotoxicity under hypoxic conditions.

## 2. Materials and Methods

### 2.1. Cells

SH-SY5y neuroblastoma cells were obtained from the Korean Cell Line Bank (KCLB No. 22266, Seoul, Republic of Korea). The cells were identified by STR profiling including D7S820, D5S818, D13S317, FGA, vWA, TPOX, and TH01 by distributor. The cells were grown in DMEM medium (Welgene, Gyeongsan-si, Republic of Korea) supplemented with 10% fetal bovine serum (FBS), penicillin G (100 units/mL), L-glutamine (2 mM), and streptomycin (100 μg/mL) at 37 °C in a humidified incubator containing 5% CO_2_. Cells were maintained within passage #14 prior to any experiments.

### 2.2. Isolation of 6,7,4’-Tryhydroxyflavanone from Dalbergia Odorifera

**Figure 1:**
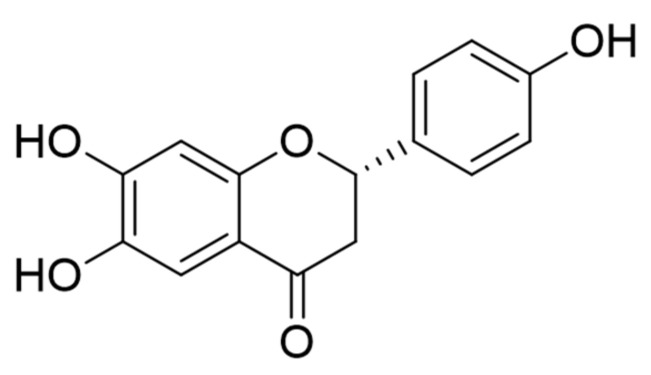
The chemical structure of 6,7,4′-trihydroxyflavanone (THF).

THF (C_15_H_12_O_5_, Figure 1) was isolated from *D. odorifera* as previously reported [15]. Briefly, *D. odorifera* was purchased from the Herbal Medicine Cooperative Association of Jeonbuk Province, Korea. Dried *D. odorifera* (20 kg) was extracted three times with 100% EtOH. The filtered EtOH extract (2.416 kg) was concentrated and partitioned with CH_2_Cl_2_. The CH_2_Cl_2_-soluble fraction (200 g) was subjected to chromatography on a silica gel column with n-hexane-EtOAc (1:0 to 0:1) to obtain five fractions (Fr. 1 to Fr. 5). Among them, Fr. 3 (120 g) was separated on a Sephadex LH-20 column with a mixture of solvents (MeOH:H_2_O = 9:1) and four fractions were obtained (Fr. 3-1 to Fr. 3-4). Of the four fractions, Fr. 3-3 (30 g) was further purified on a Sephadex LH-20 column with a mixture of solvents (EtOAc: MeOH = 4:1) and loaded on a silica gel column with a gradient mixture of solvents from 100% n-hexane to 100% EtOAc to obtain fifteen fractions (Fr. 3-3-1 to Fr. 3-3-15). Among them, Fr. 3-3-4 (120 mg) was characterized as THF by comparing the newly obtained ^1^H and ^13^C nuclear magnetic resonance (NMR, JEOL JNM-ECA 500) spectral data with a previous report [19]. The purity was determined to be 98.8% based on NMR.

### 2.3. Reagents and Antibodies

MTT powder (1-(4,5-Dimethylthiazol-2-yl)-3,5-diphenylformazan), RIPA buffer, TRIZOL reagent, CoCl_2_, DCF-DA, and Protoporphyrin IX (SnPP) were purchased from Sigma Chemical Co. (St. Louis, MO, USA). AnnexinV/PI apoptosis assay kit was obtained from BD Biosciences (San Diego, CA, USA). Antibodies against Caspase3, Caspase7, Caspase8, Caspase9, HIF1α, p53, VEGF, GLUT1, SOD, CAT, HO-1, and LaminB were purchased from Cell Signaling Technology (Danvers, MA, USA). Anti-Bcl-2, anti-β-actin, and anti-Nrf2 antibodies were obtained from Santa Cruz Biotechnology (Dallas, TX, USA). The RT PreMix kit was purchased from Enzynomics (Daejeon, Korea). SYBR Premix Ex Taq was obtained from Takara (Shiga, Japan). Nuclear and Cytoplasmic Extraction Reagents Kit (NE-PER) and ECL Western blot detection reagents were purchased from Thermo Fisher Scientific (Waltham, MA, USA).

### 2.4. MTT Assay

Seeded SH-SY5y cells (1 × 10^4^/well, 96-well plate) were treated with the indicated concentration of THF (0–40 μM) for 24 h. The supernatants were discarded and 500 μg/mL of MTT was incubated with the cells for 1 h. Supernatants were removed and generated formazan crystals were dissolved in 170 μL of DMSO. Plate was read to gain the absorbance at 540 nm and cell viability was determined by comparing with absorbance of control cells (% of control).

### 2.5. Determination of Dead Cell Population by AnnexinV Staining

Seeded SH-SY5y cells (1 × 10^4^/well, 96-well plate) were stained with 1× AnnexinV staining reagent for IncuCyte for 30 min. Then cells were treated with the indicated concentration of THF (0–40 μM) for 24 h or pre-treated with the indicated concentration of THF (0–40 μM) for 6 h and incubated with 0.5 mM CoCl_2_ for 24 h. After incubation, the intensity of AnnexinV was assessed by IncuCyte imaging system and DIC images were obtained with AnnexnV fluorescence (green). Integrated intensity of AnnexinV was determined by comparing with control cells (% of control).

### 2.6. Western Blot Analysis

SH-SY5y cells cultured in the indicated conditions were harvested for lysis in RIPA buffer with 1× phosphatase inhibitor at 4 °C for 20 min. Lysates were centrifuged at 13,500 rpm at 4 °C for 20 min and 30 to 50 μg of the lysate was separated on 8–12% sodium dodecyl sulfate polyacrylamide gel electrophoresis (SDS–PAGE) gels. Proteins were transferred onto PVDF membranes, which were then blocked with 5% skimmed milk for 1 h. After blocking, membranes were incubated with the respective primary antibodies in 3% skim milk overnight (1:1000 ratio). Excess primary antibodies were removed by washing the membrane four times with TBS-T and incubated with 0.1 μg/mL peroxidase-labeled secondary antibodies (against rabbit or mouse) for 1 h. After four washes with TBS-T, bands were detected with ECL Western blot detection reagents with an ImageQuant LAS 4000 (GE Healthcare, Chicago, IL, USA).

### 2.7. Apoptosis Assay

Apoptotic neurotoxicity of SH-SY5y cells was assessed by a double staining experiment using AnnexinV and PI. After incubation of SH-SY5y neuroblastoma cells treated with the indicated conditions, cells were suspended in 1× trypsin-ethylenediaminetetraacetic acid (EDTA) buffer. After washing with cold PBS, cells were resuspended in 100 μL of 1× binding buffer containing AnnexinV (20 μg/mL) and PI (1 μg/mL) for 15 min at RT. Stained cells were acquired on a BD FACSVerse (BD Biosciences, San Diego, CA, USA), and the population of AnnexinV^+^ cells or AnnexinV^+^/PI^+^ cells was presented in the bar graph with plots.

### 2.8. RT-PCR and Realtime Quantitative RT-PCR

Total RNA was isolated from cells treated with the indicated conditions using TRIZOL reagent and reverse transcription of the RNA to cDNA was performed. Primers used for each gene were as follows (forward and reverse primers, respectively). human *HIF1a*, 5′-ATC CAT GTG ACC ATG AGG AAA TG-3′ and 5′-TCG GCT AGT TAG GGT ACA CTT C-3′ (accession number: NM_181054); human *p53*, 5′-CCT CAG CAT CTT ATC CGA GTG G-3′ and 5′-TGG ATG GTG GTA CAG TCA GAG C-3′ (accession number: NM_000546), human *VEGF*, 5′-ACC AAG GCC AGC ACA TAG G-3′ and 5′-ACG CTC CAG GAC TTA TAC CG-3′ (accession number: NM_001171623), human *GLUT1*, 5′-TTG CAG GCT TCT CCA ACT GGA C-3′ and 5′-CAG AAC CAG GAG CAC AGT GAA G-3′ (accession number: NM_006516), human *SOD*, 5′-CTC ACT CTC AGG AGA CCA TTG C-3′ and 5′-CCA CAA GCC AAA CGA CTT CCA G-3′ (accession number: NM_000454), human *CAT*, 5′-CTT GGA ACA TTG TAC CCG CT-3′ and 5′-GTC CAG AAG AGC CTG AAT GC-3′ (accession number: NM_214301), human *GAPDH*, 5′-CGG AGT CAA CGG ATT TGG TCG TAT-3′ and 5′-AGC CTT CTC CAT GGT GGT GAA GAC-3′ (accession number: NM_001256799). For quantitative PCR analysis, amplification was performed in a DNA Engine Opticon 1 continuous fluorescence detection system (MJ Research, Waltham, MA, USA) using SYBR Premix Ex Taq. The total reaction volume was 10 μL containing 0.1 μg of cDNA and each PCR reaction was performed using the following conditions: 95 °C for 30 s, 60 °C for 30 s, and plate read for 40 cycles followed by 7 min of extension at 72 °C. Melting curve analysis was performed to characterize the dsDNA product by slowly raising the temperature (0.15 °C/s) from 60 °C to 95 °C with fluorescence data collected at 0.15 °C intervals. mRNA levels of genes were normalized with the mRNA levels of *GAPDH* and were presented as “% of maximum”. The “% of maximum” was calculated using the following equation: % of maximum = 2^−ΔΔCT^ × 100, where ΔΔCT = (CTtarget−CTgapdh) at maximum−(CTtarget−CTgapdh).

### 2.9. Reactive Oxygen Species (ROS) Measurement

SH-SY5y cells incubated with the indicated conditions were stained with 2 µM DCF-DA for 20 min in the dark. Generated fluorescence was assessed using the IncuCyte imaging system. The intensity of 2’,7’-dichlorofluorescin Diacetate (DCF-DA) was obtained from IncuCyte software and the % of maximum was calculated and presented in bar graph.

### 2.10. Detection of Nrf2 Nuclear Translocation

To detect Nrf2 nuclear translocation in SH-SY5y cells after treatment with THF, cells were incubated with the indicated concentration of THF for 1 h and collected. Nuclear extracts and cytosolic extracts were separated from the whole lysate using the NE-PER Kit. For SDS-PAGE, 20 μg of nuclear extract and 50 μg of cytosolic extract was loaded in 8% SDS gel. Nuclear extracts and cytosolic extracts were normalized with the level of LaminB and β–actin, respectively.

### 2.11. Statistics

Mean values ± SEM were evaluated from the data obtained from three independent experiments performed on separate days and presented as bar graphs. One-way ANOVA was used to determine significance (*p* value). * indicates differences from the mock-treated group or between two indicated groups considered significant at *p* < 0.05.

## 3. Results

### 3.1. THF Does Not Induce Cell Death and Apoptosis in SH-SY5y Cells

**Figure 2:**
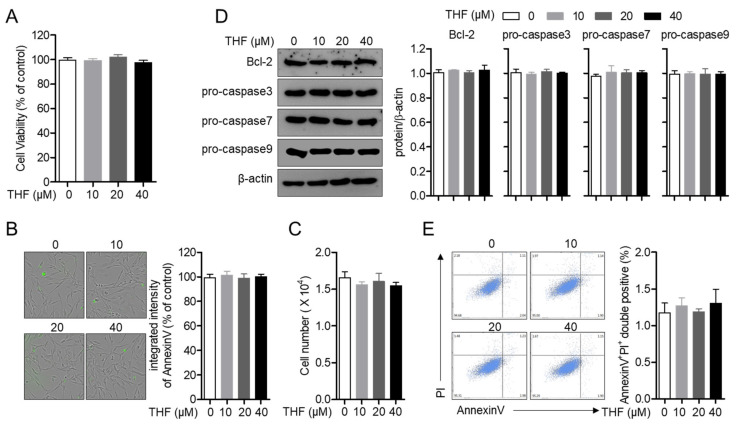
THF does not induce cell death and apoptosis in SH-SY5y cells. (**A**) SH-SY5y cells (1 × 10^4^/well, 96-well plate) were seeded and treated with the indicated concentration (0 to 40 μM) of THF for 24 h. Cell viability was assessed by MTT assay. (**B**,**C**) SH-SY5y cells (1 × 10^4^/well, 96-well plate) were stained with 1X AnnexinV staining reagent and treated with the indicated concentration (0 to 40 μM) of THF for 24 h. The intensity of AnnexinV was assessed by IncuCyte imaging system (**B**) and cell number was counted after trypan blue staining (**C**). (**D**) SH-SY5y cells (1 × 10^5^/well, 6-well plate) were treated with the indicated concentration (0 to 40 μM) of THF for 24 h and collected for Western blot analysis. Indicated proteins were detected and normalized with the level of β-actin. (**E**) Apoptosis assay was performed with SH-SY5y cells treated with the indicated concentration (0 to 40 μM) of THF for 24 h. The mean value of three experiments ± SEM is presented.

Since it has been previously reported that THF is not cytotoxic to RAW 264.7 cells [15], we first confirmed whether THF shows cytotoxicity in SH-SY5y neuronal cells. Figure 2A shows that treatment with up to 40 μM of THF does not induce cell death. The measurement of intensity of AnnexinV also exhibited that THF does not affect cell death in SH-SY5y cells (Figure 2B). The changes of cell number was not shown in cells incubated with THF up to 40 μM (Figure 2C). To investigate whether THF is associated with apoptosis in SH-SY5y cells, the changes in expression of apoptosis-related proteins after THF treatment were determined. As shown in Figure 2D, the expression of Bcl2 and caspase family, which are highly involved in apoptosis, were not altered by THF treatment. The results from the apoptosis assay also demonstrated that SH-SY5y cells treated with up to 40 μM THF did not undergo apoptotic pathway (Figure 2E). These data suggest that THF does not induce cell death and apoptosis in SH-SY5y cells.

### 3.2. THF Protects SH-SY5y Cells from CoCl_2_-Induced Cytotoxicity in Hypoxic Condition

**Figure 3:**
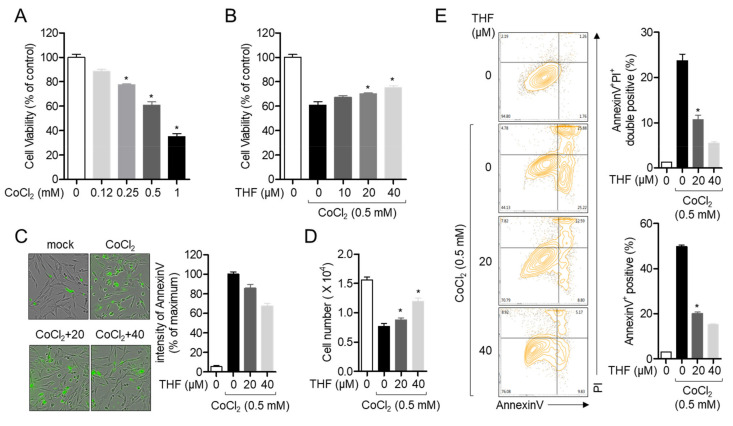
THF protects SH-SY5y cells from CoCl_2_-induced cytotoxicity in hypoxic condition. (**A**) SH-SY5y cells (1 × 10^4^/well, 96-well plate) were treated with the indicated concentration (0 to 1 mM) of CoCl_2_ for 24 h. Cell viability was assessed by MTT assay. (**B**) SH-SY5y cells (1 × 10^4^/well, 96-well plate) were pre-treated with the indicated concentration (0 to 40 μM) of THF for 6 h and treated with 0.5 mM CoCl_2_ for 24 h. Cell viability was assessed by MTT assay. (**C**,**D**) SH-SY5y cells (1 × 10^4^/well, 96-well plate) were stained with 1X AnnexinV staining reagent and pre-treated with the indicated concentration (0 to 40 μM) of THF for 6 h. After pre-treatment, cells were incubated with 0.5 mM CoCl_2_ for 24 h. The intensity of AnnexinV was assessed by IncuCyte imaging system (**C**) and cell number was counted after trypan blue staining (**D**). (**E**) Apoptosis assay was performed with SH-SY5y cells pre-treated with the indicated concentration (0 to 40 μM) of THF for 6 h and treated with 0.5 mM CoCl_2_ for 24 h. The mean value of three experiments ± SEM is presented. *p < 0.05 between mock-treated cells.

It has been widely established that treatment with cobalt chloride (CoCl_2_) induces hypoxia in neuronal cells [20]. To understand whether CoCl_2_ leads to neurotoxicity in SH-SY5y cells, MTT assay was performed. Figure 3A showed that cellular viability was reduced in dose-dependent manner. To evaluate whether pre-treatment with THF has a protective effect on the neuronal cytotoxicity induced by treatment with CoCl_2_, cell viability was assessed by MTT assay. Figure 3B shows that the viability of SH-SY5y cells pre-treated with THF was significantly restored in a dose-dependent manner compared to cells pre-treated with mock. The intensity of AnnexinV was also partially inhibited by pre-treatment with THF in CoCl_2_-induced hypoxia (Figure 3C). Cell number was also confirmed that pre-treatment with THF preserves cell viability in CoCl_2_-induced hypoxia condition (Figure 3D). To confirm if THF pre-treatment protects SH-SY5y cells from CoCl_2_-induced apoptosis, the population of AnnexinV- and PI-positive cells was determined by flow cytometry. As shown in Figure 3E, exposure to CoCl_2_ led to the apoptotic pathway in SH-SY5y cells, but THF pre-treatment partially restored the CoCl_2_-induced apoptosis in a dose-dependent manner. These data demonstrate that CoCl_2_ treatment induces neurotoxicity in SH-SY5y cells, but THF pre-treatment effectively preserves cellular death and apoptosis in a dose-dependent manner.

### 3.3. THF Blocks the Cleavage of Caspase Family in CoCl_2_-Induced Hypoxia Condition

**Figure 4:**
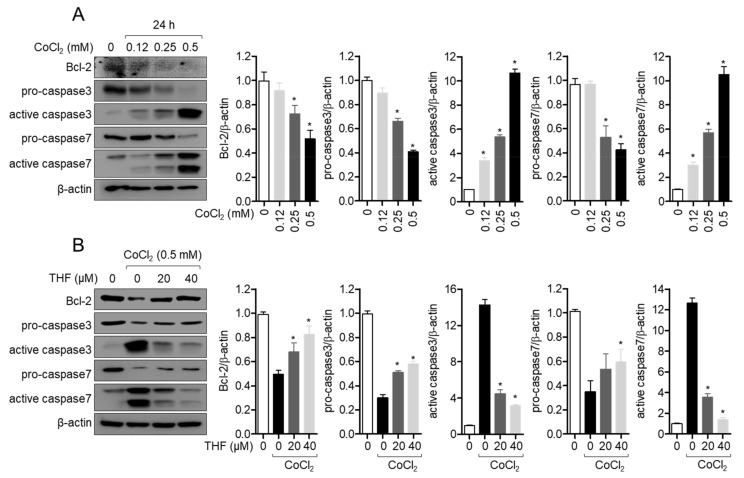
THF blocks the cleavage of caspase family in CoCl2-induced hypoxia condition. (**A**) SH-SY5y cells (1 × 10^5^/well, 6-well plate) were treated with the indicated concentration (0 to 0.5 mM) of CoCl_2_ for 24 h and collected for Western blot analysis. Indicated proteins were detected and normalized with the level of β-actin. (**B**) SH-SY5y cells (1 × 10^5^/well, 6-well plate) were pre-treated with the indicated concentration (0 to 40 μM) of THF for 6 h and treated with 0.5 mM CoCl_2_ for 24 h. Cells were collected for Western blot analysis and indicated proteins were detected. The mean value of three experiments ± SEM is presented. * *p* < 0.05 between mock-treated cells (**A**) or CoCl_2_-treated cells (**B**).

It has been evaluated that the fate of cells undergoing apoptotic pathway is tightly controlled by the expression of apoptosis-related proteins [21]. To elucidate the changes in the expression of apoptosis-related proteins after CoCl_2_ treatment of SH-SY5y cells, the expression of Bcl2 and caspase family was determined by Western blot analysis. Figure 4A shows that CoCl_2_ treatment downregulates the expression of Bcl2 and cleavage of caspase3 and 7 in SH-SY5y cells in a dose-dependent manner. To validate whether THF pre-treatment blocks reduction of the apoptosis-related proteins in CoCl_2_-induced hypoxic condition, Western blot analysis was performed. As shown in Figure 4B, THF pre-treatment partially restored the suppressed expression of Bcl2 and led to cleavage of caspase3 and caspase7 by CoCl_2_ treatment. These data suggest that THF pre-treatment preserves the expression of anti-apoptotic proteins but suppresses the active caspase family in CoCl_2_-induced hypoxic condition.

### 3.4. THF Inhibits CoCl_2_-Induced Hypoxia-Related Genes in SH-SY5y Cells

**Figure 5:**
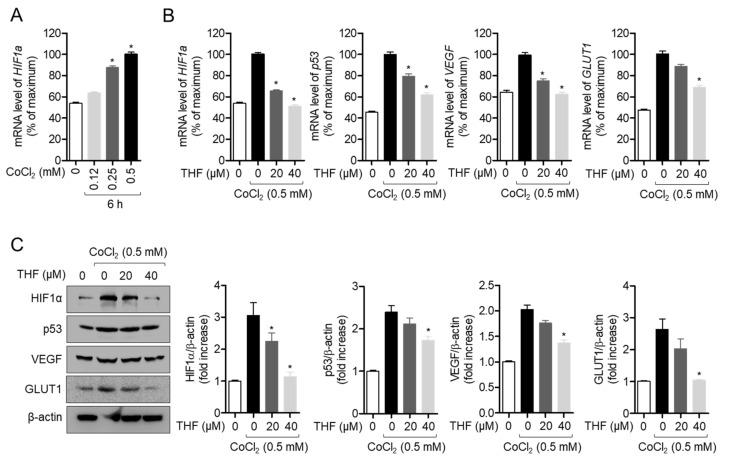
THF inhibits CoCl_2_-induced hypoxia-related genes in SH-SY5y cells. (**A**) SH-SY5y cells (1 × 10^5^/well, 6-well plate) were treated with the indicated concentration (0 to 0.5 mM) of CoCl_2_ for 6 h and collected for the real-time PCR analysis. The mRNA level of *HIF1a* was measured and normalized with the level of *GAPDH*. (**B**) SH-SY5y cells (1 × 10^5^/well, 6-well plate) were pre-treated with the indicated concentration (0 to 40 μM) of THF for 6 h and treated with 0.5 mM CoCl_2_ for 6 h. The mRNA level of the indicated genes were measured and normalized with the level of *GAPDH*. (**C**) SH-SY5y cells (1 × 10^5^/well, 6-well plate) were pre-treated with the indicated concentration (0 to 40 μM) of THF for 6 h and treated with 0.5 mM CoCl^2^ for 24 h. Cells were collected for Western blot analysis and indicated proteins were detected. The mean value of three experiments ± SEM is presented. * *p* < 0.05 between mock-treated cells (**A**) or CoCl_2_-treated cells (**B**,**C**).

To investigate the underlying mechanism of how THF pre-treatment protects the cells from neurotoxicity induced by CoCl_2_ treatment in SH-SY5y cells, we first tested whether THF pre-treatment blocks hypoxia induced by CoCl_2_ treatment. As shown in Figure 5A, CoCl_2_ exposure induced the mRNA level of *HIF1a*, a marker of hypoxia, in a dose-dependent manner. Under hypoxic condition, we found that THF pre-treatment significantly inhibited the induction of *HIF1a* by treatment with 0.5 mM CoCl_2_ (Figure 5B). We also checked if THF pre-treatment reduced the mRNA levels of hypoxia-related genes, including *p53*, *VEGF*, and *GLUT1*. Quantitative RT-PCR results showed that THF pre-treatment significantly suppressed the induction of *p53*, *VEGF*, and *GLUT1* expression (Figure 5B). The regulatory effects of THF pre-treatment on the induction of hypoxia-related genes were also confirmed by Western blot at the protein level (Figure 5C). These results clearly demonstrate that THF pre-treatment attenuates the CoCl_2_-induced hypoxic condition in SH-SY5y cells.

### 3.5. THF Attenuates the CoCl_2_-Induced Oxidative Stress in SH-SY5y Cells

**Figure 6:**
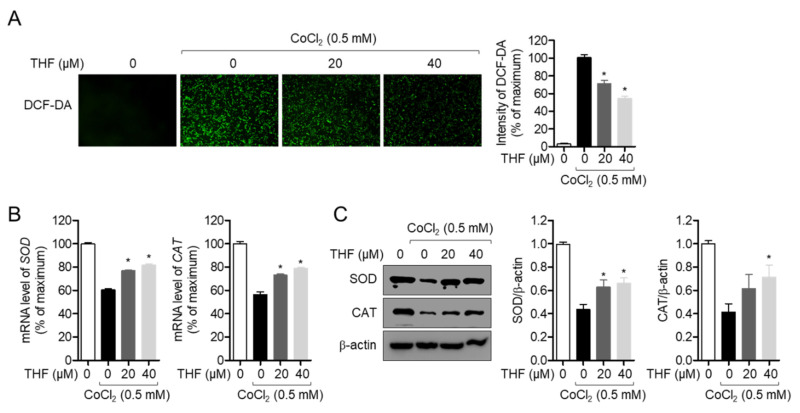
THF attenuates the CoCl_2_-induced oxidative stress in SH-SY5y cells. (**A**) SH-SY5y cells (1 × 10^4^/well, 96-well plate) were pre-treated with the indicated concentration (0 to 40 μM) of THF for 6 h and treated with 0.5 mM CoCl_2_ for 24 h. After incubation with 2 μM of DCF-DA for 20 min in dark, generated ROS were detected by IncuCyte imaging system. (**B**) SH-SY5y cells (1 × 10^5^/well, 6-well plate) were pre-treated with the indicated concentration (0 to 40 μM) of THF for 6 h and treated with 0.5 mM CoCl_2_ for 6 h. The mRNA level of the indicated genes were measured and normalized with the level of *GAPDH*. (**C**) SH-SY5y cells (1 × 10^5^/well, 6-well plate) were pre-treated with the indicated concentration (0 to 40 μM) of THF for 6 h and treated with 0.5 mM CoCl_2_ for 24 h. Cells were collected for Western blot analysis and indicated proteins were detected. The mean value of three experiments ± SEM is presented. * *p* < 0.05 between CoCl_2_-treated cells.

One of the well-defined cytotoxic mechanisms of hypoxia is the induction of oxidative stress [22]. To explore whether THF pre-treatment is associated with the inhibition of CoCl_2_-induced oxidative stress, we assessed the ROS generation in THF pre-treated and CoCl_2_-exposed SH-SY5y cells. Figure 6A revealed that enhanced ROS generation by CoCl_2_ exposure was significantly suppressed by THF pre-treatment in a dose-dependent manner. Since oxidative stress, including ROS generation by CoCl_2_, is highly involved in the reduced expression of SOD and CAT, which are antioxidant proteins, we also determined if THF pre-treatment preserves them in CoCl_2_-induced hypoxia. Quantitative RT-PCR analysis showed that the mRNA levels of *SOD* and *CAT* in CoCl_2_-exposed SH-SY5y cells were downregulated, which was restored by THF pre-treatment (Figure 6B). It was also confirmed on the protein level by Western blot analysis that the expressions of SOD and CAT are preserved by THF pre-treatment (Figure 6C). These results suggest that THF pre-treatment mitigates the CoCl_2_-induced oxidative stress by restoring the expression of antioxidant proteins.

### 3.6. THF Promotes HO-1 Expression by Leading Nrf2 Translocation in SH-SY5y Cells

**Figure 7:**
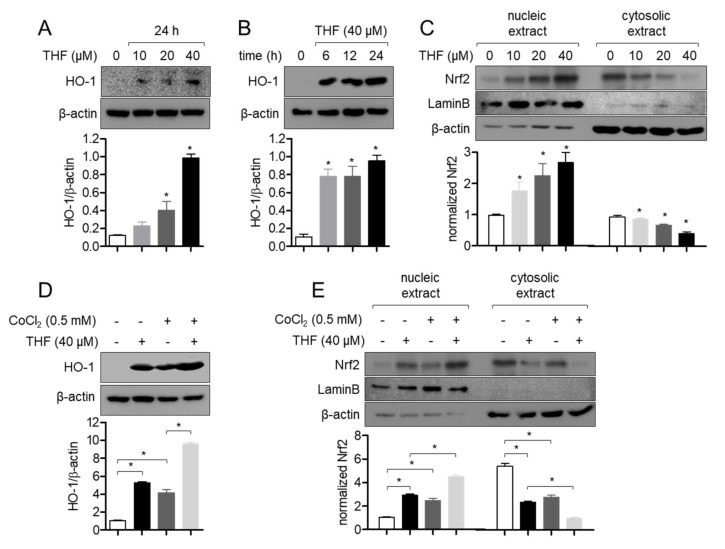
THF promotes HO-1 expression by leading Nrf2 translocation in SH-SY5y cells. (**A**,**B**) SH-SY5y cells (1 × 10^5^/well, 6-well plate) were treated with the indicated concentration (0 to 40 μM) of THF for the indicated time (0 to 24 h) and collected for Western blot analysis. The level of HO-1 was detected and normalized with the level of β-actin. (**C**) SH-SY5y cells (1 × 10^5^/well, 6-well plate) were treated with the indicated concentration (0 to 40 μM) of THF for 1 h and collected for Western blot analysis. Nucleic extract was separated from whole lysate by using NE-PER kit. The expression of Nrf2 was detected from nucleic extract and cytosolic extract. (**D**) SH-SY5y cells (1 × 10^5^/well, 6-well plate) were pre-treated with 40 μM of THF for 6 h and cultured with 0.5 mM CoCl_2_ for 24 h. The level of HO-1 was detected and normalized with the level of β-actin. (**E**) SH-SY5y cells (1 × 10^5^/well, 6-well plate) were treated with 40 μM of THF for 1 h and cultured with 0.5 mM CoCl_2_ for 1 h. Nucleic extract was separated from whole lysate by using NE-PER kit. The translocation of Nrf2 was detected from nucleic extract and cytosolic extract. The mean value of three experiments ± SEM is presented. * *p* < 0.05 between mock-treated cells (**A**–**C**) or two cells (**D**,**E**).

HO-1 has been widely reported as an important product of the antioxidant signaling pathway [23]. To evaluate whether THF treatment is associated with HO-1 induction, we detected the expression of HO-1 in THF-treated SH-SY5y cells in a dose-dependent manner. Figure 7A clearly demonstrates that the expression of HO-1 is induced by THF treatment. The time-dependent experiment showed that the expression of HO-1 was inducible in cells treated with 40 μM THF for 6 h (Figure 7B). Since the Nrf2 pathway is known to be a major signaling pathway for the induction of HO-1, we checked whether treatment with THF leads to the nuclear translocation of Nrf2 into the nucleus in SH-SY5y cells. Figure 7C shows that Nrf2 is translocated into the nucleus by a dose-dependent THF treatment in SH-SY5y cells. Besides, we performed the Western blot assay to explore whether both CoCl_2_ and THF affect to Nrf2 nuclear translocation and HO-1 expression. As shown in the Figure 7D, HO-1 expression is induced by exposure to CoCl_2_ but pre-treatment with THF promotes more HO-1 expression compared to CoCl_2_ exposure only. We also found that exposure to CoCl_2_ induces the Nrf2 nuclear translocation but pre-treatment with THF boosts it in SH-SY5y cells (Figure 7E). These data suggest that THF treatment enhances the expression of HO-1 through Nrf2 nuclear translocation and exposure to CoCl_2_ stimulates the defense mechanism inside cells but pre-treatment with THF improves defense pathway against toxicity including CoCl_2_ in SH-SY5y cells.

### 3.7. Enhancement of HO-1 by THF Pre-treatment Protects SH-SY5y Cells from CoCl_2_-Induced Neurotoxicity in Hypoxic Condition

**Figure 8:**
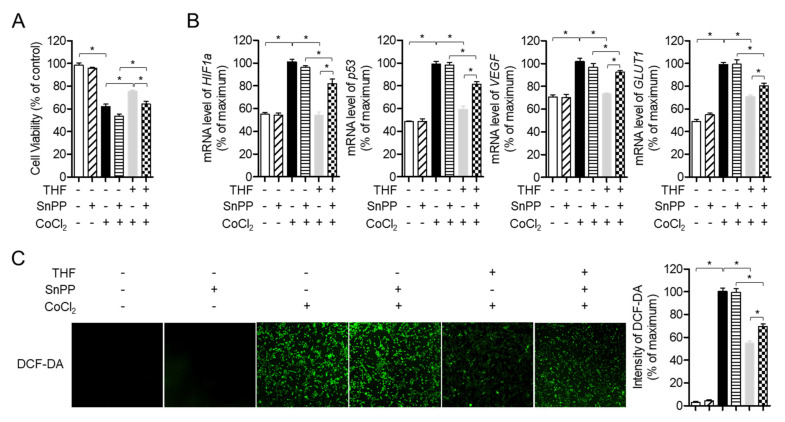
Enhancement of HO-1 by THF pre-treatment protects SH-SY5y cells from CoCl_2_-induced neurotoxicity in hypoxic condition. (**A**) SH-SY5y cells (1 × 10^4^/well, 96-well plate) were pre-treated with 20 μM SnPP for 1 h and then the indicated concentration (0 to 40 μM) of THF for 6 h. After treatment with 0.5 mM CoCl_2_ for 24 h, cell viability was assessed by MTT assay. (**B**) SH-SY5y cells (1 × 10^5^/well, 6-well plate) were pre-treated with 20 μM SnPP for 1 h and then the indicated concentration (0 to 40 μM) of THF for 6 h. After treatment with 0.5 mM CoCl_2_ for 6 h, cells were harvested for the isolation of total RNA. The mRNA level of the indicated genes were measured and normalized with the level of *GAPDH*. (**C**) SH-SY5y cells (1×10^4^/well, 96-well plate) were pre-treated with 20 μM SnPP for 1 h and then the indicated concentration (0 to 40 μM) of THF for 6 h. After treatment with 0.5 mM CoCl_2_ for 24 h, cells were incubated with 2 μM of DCF-DA for 20 min in dark. Generated ROS were detected by IncuCyte imaging system. The mean value of three experiments ± SEM is presented. * *p* < 0.05 between two indicated groups.

Since the expression of induced HO-1 by antioxidants has been reported to protect cells against cytotoxic conditions, including hypoxia, we investigated whether HO-1 induction by THF treatment is involved in the protective role of THF under hypoxic conditions. To remove the cytoprotective effect of HO-1 induced by THF pre-treatment, cells were pre-treated with 20 μM SnPP to inhibit the activity of HO-1, then the cell viability was assessed. Figure 8A shows that pre-treatment with SnPP significantly mitigates the protective effect of THF in SH-SY5y cells. SH-SY5y cells pre-treated with SnPP also revealed undiminished mRNA levels of hypoxia-related genes, including *HIF1a*, *p53*, *VEGF*, and *GLUT1* (Figure 8B). To confirm whether pre-treatment with SnPP removes the antioxidative effect of THF in CoCl_2_-induced hypoxia, generated ROS were measured in SH-SY5y cells pre-treated with SnPP and THF and exposed to CoCl_2_. Figure 8C shows that the suppressive effect of THF pre-treatment on ROS generation was mitigated in SH-SY5y cells. Interstingly, treatment with 20 μM SnPP does not affect to cell viability, mRNA level of hypoxia-related genes and ROS production. These data demonstrated that induction of HO-1 expression by THF pre-treatment protects SH-SY5y cells from cytotoxicity induced by CoCl_2_ treatment.

## 4. Discussion

It has been widely studied that the expression of VEGF and GLUT1 are regulated by HIF1α under hypoxic conditions. The biological function of VEGF has been reported to promote angiogenesis and increase the permeability of blood vessels under hypoxic conditions [24,25]. GLUT1 is a part of glucose transporter family that is located on the cell membrane and induces glucose transport to the cells. Several previous studies have demonstrated that cells induce the expression of VEGF and GLUT1 to absorb more oxygen in situations where oxygen is limited [7,8,9]. In addition, it has been shown that HIF1α binds to a specific sequence in target genes of hypoxia-responsive promoters, including *p53*, *VEGF*, and *GLUT1*, depending on the concentration of oxygen [26]. In this study, we investigated whether CoCl_2_-induced hypoxia induces the expression of HIF1α and THF pre-treatment suppresses this increment in SH-SY5y cells (Figure 5). We also showed that THF pre-treatment reduced the expression of *VEGF* and *GLUT1* at the mRNA and protein levels. Results from the inhibitor assay confirmed that induction of HO-1 by THF pre-treatment plays a critical role in cytoprotection under hypoxic conditions (Figure 8). These results suggest that pre-treatment with THF indirectly regulates the expression of HIF1α via promotion of the HO-1/Nrf2 pathway rather than direct regulation in vitro. Further studies should include whether THF is directly involved in the transcription of *HIF1a*, *p53*, *VEGF*, and *GLUT1* genes by performing EMSA assay.

To maintain normal cellular signaling response, ROS generation is tightly regulated in brain tissue through the expression of antioxidant enzymes, including SOD and CAT. In particular, strategies to reduce oxidative stress in brain tissue have been considered promising for the development of therapeutics for neurodegenerative diseases. One of the main factors inducing oxidative stress in the brain is the limited concentration of oxygen that causes a hypoxic environment. Excessive ROS generated in hypoxic conditions leads to apoptosis, DNA damages, and cell death. We confirmed whether exposure to CoCl_2_ augments ROS generation in SH-SY5y cells, but THF pre-treatment effectively suppresses generated ROS (Figure 6A). In addition, the expression of SOD and CAT was significantly upregulated in a THF dose-dependent manner (Figure 6B,C). Figure 8C confirmed that HO-1 induced by THF pre-treatment is highly involved in the regulatory role of THF in the generation of ROS under hypoxic conditions. These data suggest that promotion of HO-1 expression by THF pre-treatment effectively protects neuronal cells from neurotoxicity induced by hypoxic condition.

Anti-apoptotic or cytoprotective effects of flavonoids have been widely elucidated. Quercetin, the most studied flavonoid, has been investigated that it partially blocks H_2_O_2_-induced apoptosis through the regulation of activator protein 1 (AP-1) transcription factor [27]. In particular, flavanone compounds have been studied as anti-apoptotic activity. Treatment with hesperetin and its metabolites, 5-nitro-hesperetin has shown a protective effect on neuronal cell death by modulation of ERK/PI3K pathway and naringenin possesses an anti-apoptotic activity in ischaemic stroke damage via Nrf2/HO-1 signaling pathway [28,29]. In the present study, we explored the anti-apoptotic effect of THF, one of flavanone compounds, in CoCl_2_-induced hypoxia condition through induction of Nrf2/HO-1 cytoprotective pathway. Our study suggests that a flavanone family including naringenin, hesperetin and THF has a potential to be developed as a source of therapeutic for neurodegenerative diseases.

## 5. Conclusions

In this study, we evaluated the cytoprotective effect of THF on CoCl_2_-induced neurotoxicity by promoting the HO-1/Nrf2 pathway. We showed that THF pre-treatment effectively enhanced the expression of HO-1 through the Nrf2 pathway in SH-SY5y cells and induced HO-1 suppresses the expression of hypoxia-related genes induced by CoCl_2_ treatment. This reduced hypoxic condition by THF pre-treatment mitigates oxidative stress and leads to protection of SH-SY5y cells from neurotoxicity by CoCl_2_ treatment.

## Data Availability

The data presented in this study are available on request from the corresponding author.
